# The initial deficiency of protein processing and flavonoids biosynthesis were the main mechanisms for the male sterility induced by SX-1 in *Brassica napus*

**DOI:** 10.1186/s12864-018-5203-y

**Published:** 2018-11-07

**Authors:** Luyun Ning, Zhiwei Lin, Jianwei Gu, Lu Gan, Yonghong Li, Hao Wang, Liyun Miao, Libin Zhang, Baoshan Wang, Maoteng Li

**Affiliations:** 1 Department of Biotechnology, College of Life Science and Technology, Huazhong University of Science and Technology, Wuhan, 430074 China; 2 Hybrid Rape Research Center of Shaanxi Province, Shaanxi Rapeseed Branch of National Centre for Oil Crops Genetic Improvement, Yangling, 712100 China; 3 College of Life Science, Shandong Normal University, Jinan, 250000 China; 4 Hubei Collaborative Innovation Center for the Characteristic Resources Exploitation of Dabie Mountains, Huanggang Normal University, Huanggang, 438000 China

**Keywords:** *Brassica napus*, Male sterility, Chemical hybridization agent, Proteome, Transcriptome, miRNA, Tryphine, Protein processing, Flavonoids biosynthesis, Transcript factor

## Abstract

**Background:**

Rapeseed (*Brassica napus*) is an important oil seed crop in the Brassicaceae family. Chemical induced male sterility (CIMS) is one of the widely used method to produce the hybrids in *B. napus*. Identification of the key genes and pathways that involved in CIMS were important to understand the underlying molecular mechanism. In the present report, a multi-omics integrative analysis, including of the proteomic, transcriptomic and miRNAs, combined with morphological and physiological analysis were conducted.

**Results:**

Earlier degeneration of the tapetosomes and elaioplasts, aberrantly stacking in tapetal cells and incompletely deposition in tryphine of pollen wall were observed in chemical hybridization agent (CHA) of SX-1 treated *B. napus* through SEM and TEM analysis. It was revealed that the deficiencies in protein processing in endoplasmic reticulum (ER) and flavonoids biosynthesis were occurred at early stage in the SX-1 treated materials. Subsequently, plant hormone signal transduction, biosynthesis of amino acids, fatty acids and steroid in anther at later stages were identified down-regulated after SX-1 treatment. 144 transcript factors (TFs) were also indentified to down-regulated at early stage, which suggested the early regulation in anther and pollen wall development were disordered in CHA treated *B. napus*. In addition, 7 important miRNAs were identified and 2 of the predicted target genes of miRNAs were Rf-like genes.

**Conclusions:**

Taken together, an interaction network of candidate genes and the putative metabolism pathways were constructed based on the multi-omics integrative analysis, it provided a new insight into the male sterility induced by CHA of SX-1 in *B. napus*.

**Electronic supplementary material:**

The online version of this article (10.1186/s12864-018-5203-y) contains supplementary material, which is available to authorized users.

## Background

Rapeseed (*Brassica napus*) is an important oil seed crop not only for its edible oil but also for industrial materials such as biodiesel and lubricants. Rapeseed has strong heterosis, and the hybrids could largely enhance the production up to over 30% [1]. Hybrid breeding in rapeseed is highly depended on the male sterility, which mainly including of cytoplasmic male sterility (CMS), genic male sterility (GMS) and chemical induced male sterility (CIMS) [2]. However, to select the stable CMS and GMS lines would take a long time and easily influenced by the environment such as temperature [3, 4] and photoperiod and humidity [4, 5].

CIMS is generated by chemical hybridization agents (CHA) and it could induce male sterility with not affect to the pistil [6], it’s theoretically that almost any cultivar could be used as the female parent after CHA treatment. In addition, CIMS doesn’t need prior selecting to match maintainer and restorer lines like CMS or remove the fertile plants from the female parents as half offspring of GMS are fertile [7]. Therefore, CHAs are getting more and more attention and have been widely applied, such as tribenuron-methyl (TM) [8, 9], monosulphuron ester sodium (MES) [10, 11] in *B. napus* and SQ-1 in *Triticum aestivum* [12, 13]. In MES induced male sterility, genes involved in cellular transport, lipid and carbohydrate metabolism were differentially expressed [11]. It has been proved that TM was polar-transported to anthers after spraying on leaves, then resulted in branched-chain amino acid (BCAA, including of leucine, isoleucine and valine) starvation by anther-specific acetolactate synthase (*ALS*) inhibition and autophagic cell death in anthers [9]. While the mutation of *ALS* with a substitution of proline with leucine or serine at position 197 will gain the resistance of TM and exhibit normal male fertility [8]. In SQ-1 treated wheat, premature tapetum degraded in advance and the defective in carbohydrate metabolism and oxidase pathway were the reason for the occurrence of pollen sterile [12, 13].

Tapetum is located in the innermost cell layer of anther, it began to degenerate from pollen mitotic stage and provide young microspores nutrients and then totally degrade until the maturation of pollen [14–17]. Previous studies have showed male sterility occurred when the unnormal tapetum development was occurred [18–20]. Pollen surface is covered by a sculpted wall, including intine, exine and tryphine (pollen coat), and the exine contains a highly stable and recalcitrant biopolymer called sporopollenin [17, 21]. The tryphine covers the patterned sporopollenin framework and fills the cavities of the exine with complex lipids, flavonoids, wax esters, proteins and so on, which protects the released pollen grain from various biotic and abiotic stress, and plays important roles in the adhesion and recognition ability in the pollination [22–24].

Here, we have developed one CHA named SX-1 (national patent No: ZL 03105389.0), which is effective, low poisonousness and suitable for various *B. napus* lines for breeding [25]. The major ingredient of SX-1 is sulfonylurea, which targets the catalytic subunit (*CSR1*) of *ALS*, an enzyme in the first step of the BCAA biosynthesis. The utilization effect of SX-1 on *B. napus* was very high, when treated with different concentration of SX-1 in two ecological regions, the sterile rates of cultivar “YD66A” could up to 98.11 and 100%, respectively, and the sterile rates of cultivar “Cn10” were all 100% [26]. Some excellent hybrid cultivars of *B. napus*, such as Zayou 66 and Qinyou 33 have been successfully cultivated by using the SX-1 treatment [26]. SX-1 also has been successfully used in pollen control in CMS line breeding [27]. However, the mechanism of the male sterility induced by SX-1 in *B. napus* is still unknown. In the present study, the CIMS induced by SX-1 in *B. napus* was analyzed by proteomic, transcriptomic and miRNAs analysis, combined by additional morphological and physiological analysis. The related interaction network and a putative metabolism pathway based on the candidate genes corresponding to DEPs, DEGs, DE-miRNAs were constructed. Finally, we proposed a model and provided new insights into the male sterility induced by SX-1 in *B. napus.*

## Results

### Morphological and cytological comparison of anthers and pollens in CHA treated materials and control floral buds

**Fig. 1:**
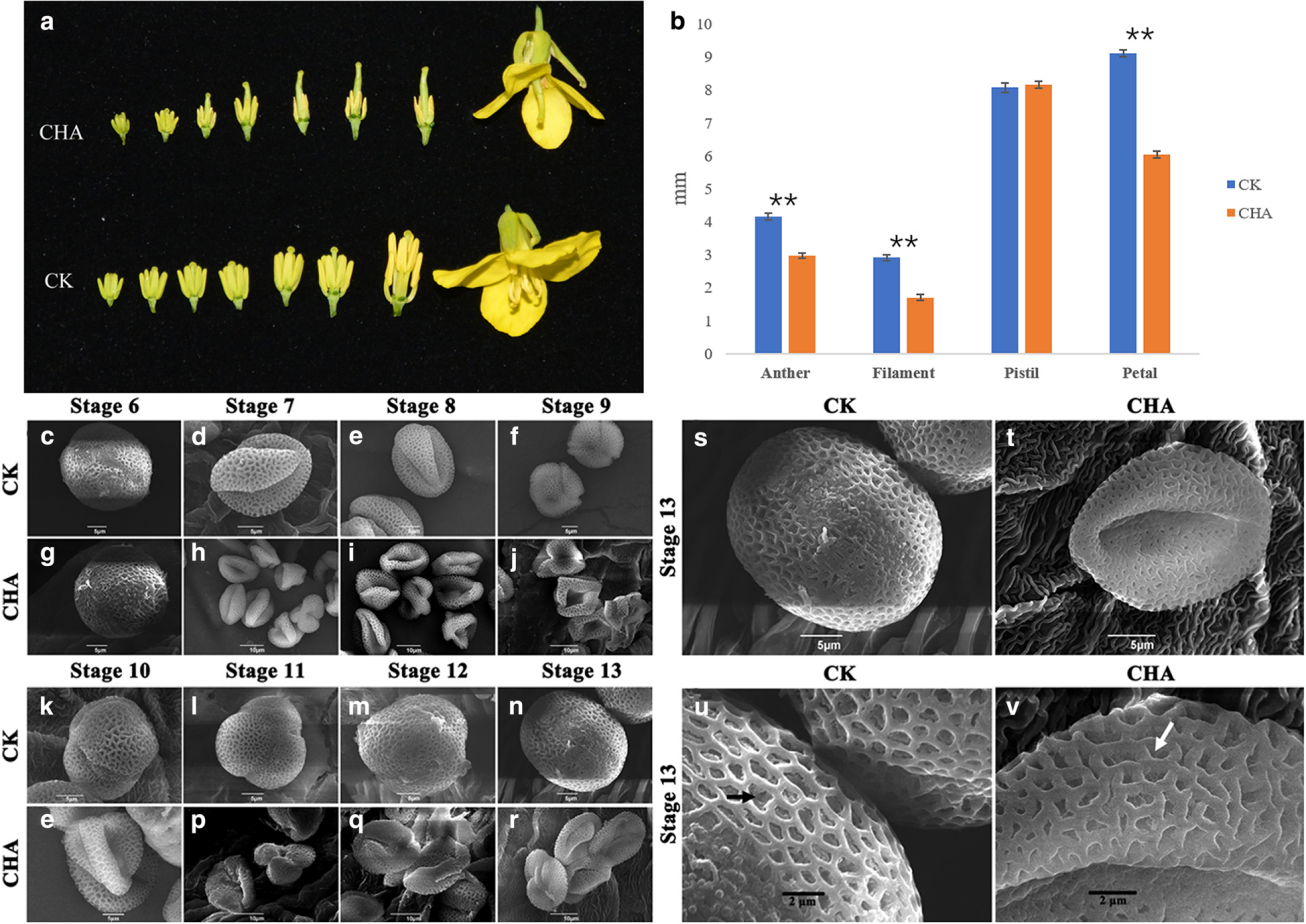
Phenotypic characterization of fertile and sterile buds and the dynamic SEM analysis of pollen grains. **a**, CK, control materials. CHA, the materials treated with SX-1. **b**, Length of anther, filament and pistil, and width of petal in opened flowers (Student’s t test, *P < 0.05, **P < 0.01). **c**-**f**, **k**-**n**, **s** and **u**, The pollen grains in control. **g**-**j**, **o**-**r**, **t** and **v**, The pollen grains in CHA treated materials. Scale bar in h-j, p-r,10 μm. Scale bar in c-g, k-o, s and t, 5 μm. Scale bar in u and v, 2 μm

It was revealed that the petals of control flowers were significantly wider than those in CHA treated materials, and the anthers and filaments in flowers with no CHA treatment were obviously longer than those in CHA treated materials (Fig. 1a and b). The anthers in sterile buds gradually shrivel from the middle stage and didn’t split and release pollen at last, while the pistil with CHA treatment was normal.

To further understand the ultrastructure difference of anthers between CHA treated materials and control, SEM and TEM observation for pollens were conducted. For SEM analysis, no distinct differences were observed in pollens before stage 7 between CHA treated materials and control. The pollen grains that treated with CHA were appeared loss of inclusion, and presented shriveled compared to control at stage 8 (Fig. 1e and i). In addition, the tryphine of control was fulfilled with some substances, while it does not exist on CHA (Fig. 1u and v).

**Fig. 2:**
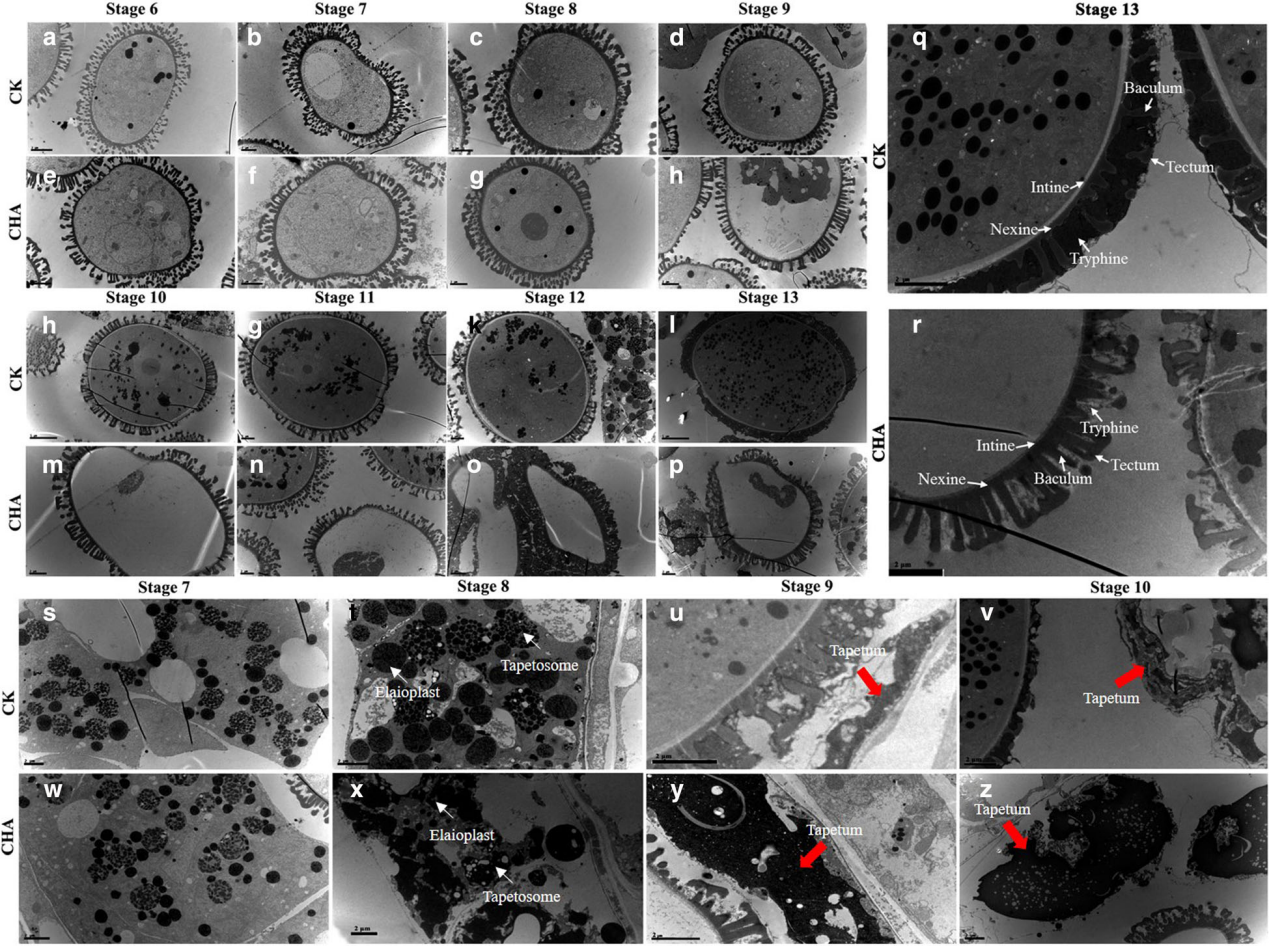
The dynamic TEM analysis of the developmental pollen grains and tapetum cell of *B. napus*. **a**-**d** and **i**-**l**, The developing pollen grains of control groups. **e**-**h** and **m**-**p**, The developing pollen of CHA treated materials. **q**, Pollen coats in control groups. **r**, Enlarged pollen coats in CHA treated materials. **s**-**v**, Tapetum in control groups. **w**-**z**, Tapetum in CHA treated materials. Red arrow, the degraded tapetal cells. Scale bar, 2 μm

For TEM analysis, no obvious difference was observed before stage 8 between the control (Fig. 2a-c) and CHA treated materials (Fig. 2e-g), the abnormal development was observed in most of CHA pollen grains from stage 9, including plasmolysis phenomenon, deletion of organelles and cytoskeleton, and totally empty pollens at last (Fig. 2m-p). What’s more, the tryphine was not deposited completely in the pollen wall compared to normal pollens (Fig. 2q and r). In addition, no obvious difference was observed in the tapetum before stage 7, but the tapetosomes and elaioplasts disassembled earlier in tapetal cells of CHA treated materials than those in control until stage 8 (Fig. 2t and x). The thickness of CHA tapetal cells were 10.3 ± 0.21 μm (Fig. 2u and v), while only 1.02 ± 0.12 μm in control (Fig. 2y and z), which implied that the tapetal cells were undergoing normal PCD process in control, while this process was delayed with the tapetal cells stacked together in CHA treated materials.

### Comparative proteomic analysis between the anthers that treated with CHA and control

**Fig. 3:**
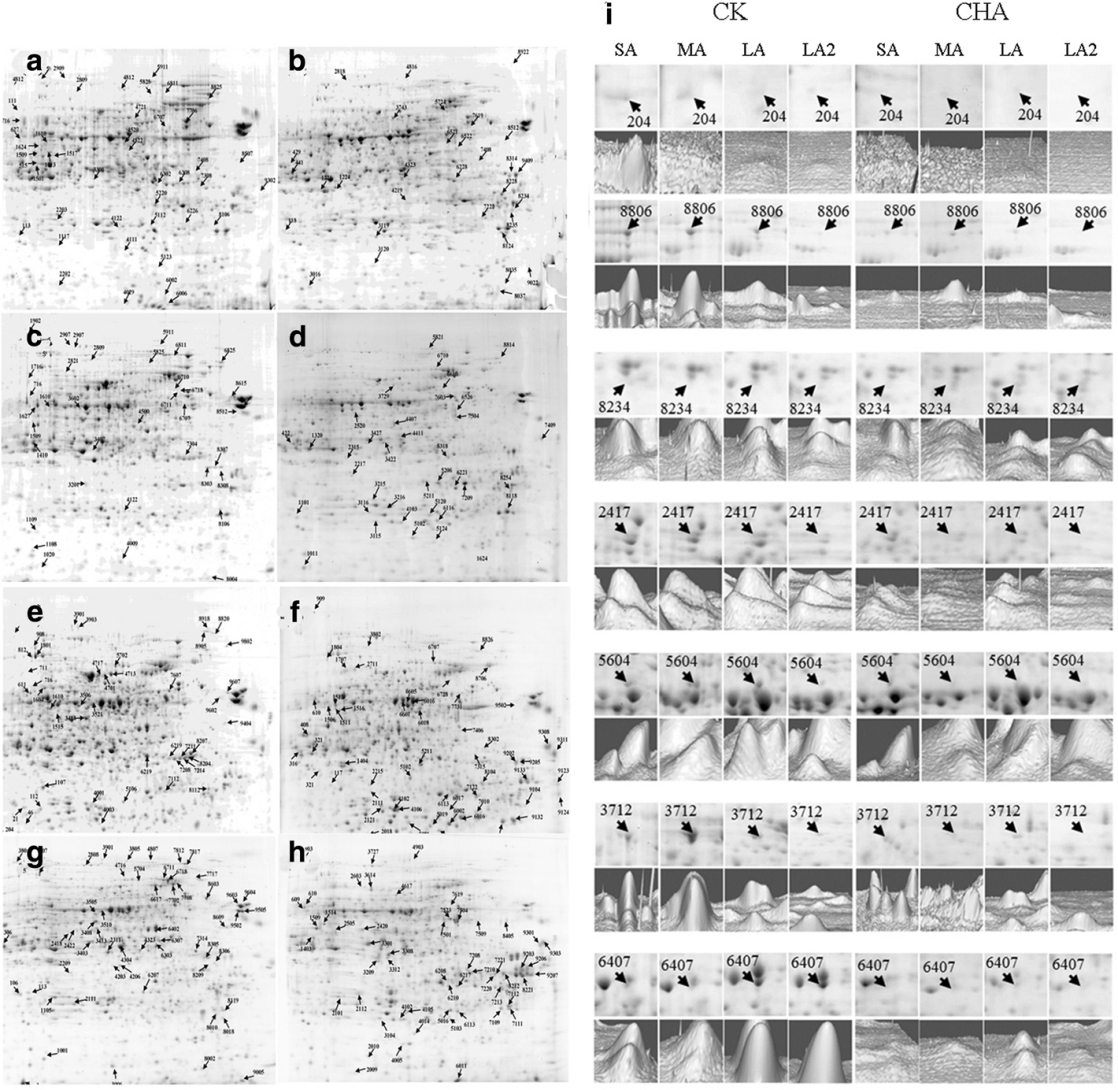
Representative 2-DE images of control and CHA groups and the enlarged area of 2-DE gel of some representative DEPs. **a**, **c**, **e** and **g**, Representative 2-DE images of control at SA, MA, LA and LA2 stage respectively. **b**, **d**, **f** and **h**, Representative images of CHA treated materials at SA, MA, LA and LA2 stage respectively. **i**, The enlarged area of 2-DE of some representative DEPs

In order to detect differentially expressed proteins (DEPs) at different stages, two-dimensional electrophoresis (2-DE) with three biological triplicates were conducted for total protein of anthers from CHA treated materials and control. As a result, 1 024, 723, 1 207 and 1 120 protein spots at SA (stage 6–7), MA (stage 8–9), LA (stage 10–11) and LA2 (stage 12–13) of control, and 847, 409, 940 and 771 protein spots at SA, MA, LA and LA2 stage of CHA treated materials were detected, respectively (Fig. 3). Compared to control, 87, 25, 74 and 60 increased expression spots were identified that changed in abundance over two-fold and exceeded the least significant difference (*p* < 0.05) at SA, MA, LA and LA2 stages respectively, and the decreased spots were 130, 220, 329 and 366, respectively.

One hundred nineteen DEPs that showed difference expression more than two stages were chosen for MALDI-TOF-MS-MS analysis and 101 DEPs were successfully identified (Additional file 1). These DEPs were grouped into 16 categories, including protein metabolism (29.7%), energy metabolism (14.9%), amino acid metabolism (8.9%), defense (6.9%), carbohydrate metabolism (5.9%), and so on. The enlarged area of 2-DE gel of some representative DEPs were showed in Fig. 3i, including protein tapetum 1 (spot 204; ATA1, AT3G42960), which was involved in amino acid metabolism, protein disulfide isomerase precursor-like (spot 8806; AT5G60640), cysteine protease (spot 8234; AT5G60360) and carboxypeptidase (spot 5604; AT3G45010) which were related to protein metabolism, chalcone synthase 3 protein (spot 2417; AT1G02050) which was involved in metabolism of terpenoids and polyketides, auxin responsive-like protein (spot 3712; AT5G13370) which was related to signal transduction, and one cytoskeleton protein, Actin-12 (spot 6407; AT3G46520). All of these proteins were down-regulated at CHA treated anther.

**Fig. 4:**
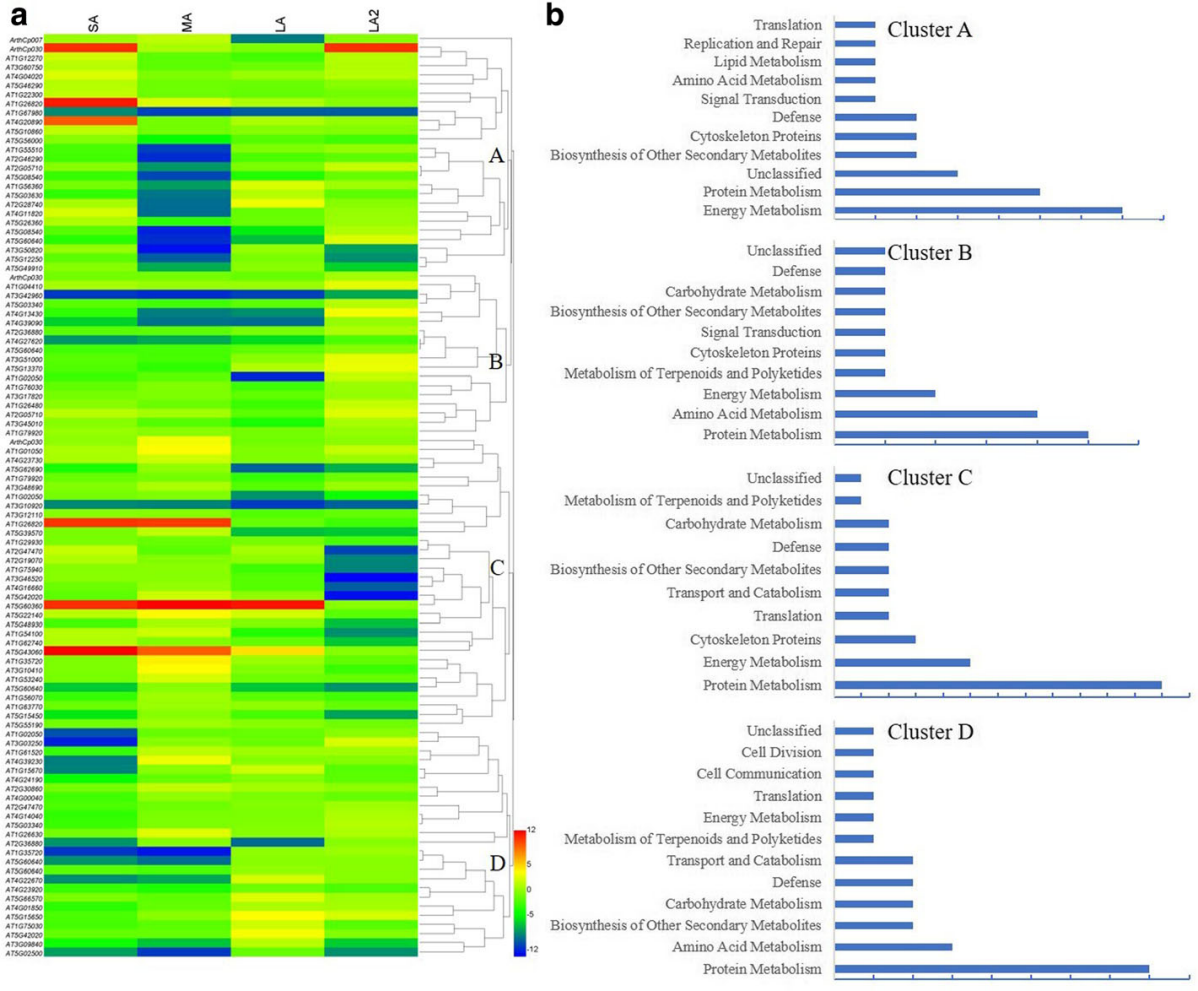
Protein expression profiles of the DEPs between CHA treated materials and control. **a**, The hierarchical clustering of the quantitative change proteins. **b**, Protein functional classification of four clusters

To further facilitate the biological interpretation of the identified DEPs, the hierarchical clustering analysis of the quantitative DEPs were conducted and four major clusters were recognized (Fig. 4a), which revealed that the DEPs between fertile and sterile anther at different development stages showed great changes in their expression patterns. Further research indicated that Cluster A accounted for 26 proteins and most of them down-regulated at MA stage, with energy metabolism and protein metabolism dominant (Fig. 4b), such as transketolase-like protein (spot 5702; AT3G60750) and chloroplast HSP70–1 (spot 8811; AT5G49910). Cluster B included 18 proteins with the down-regulation at MA and LA stage, with protein metabolism and amino acid metabolism dominant (Fig. 4b), for example, serine carboxypeptidase-like 48 (spot 5604; AT3G45010), s-adenosylmethionine synthetase (spot 627; AT2G36880), and so on. Cluster C, the largest cluster, almost decreased expressed at LA2 stage, represented 32 proteins with protein metabolism and energy metabolism proteins as the dominant classification (Fig. 4b), for instance, HSP70 (spot 6903; AT1G79920) and soluble inorganic pyrophosphatase (spot 5106; AT1G01050). Cluster D, accounted for 25 proteins that almost showed significant down-regulation at YB stage, in which the protein metabolism and amino acid metabolism were the majority classification (Fig. 4b), such as protein disulfide-isomerase A6 (spot 2314; AT2G47470) and glutathione transferase (spot 1101; AT2G30860).

### Comparative transcriptomic analysis between the anthers of CHA treated materials and control

Comparative transcriptomics was conducted to identify genes associated with male sterility induced by SX-1, three biological replicates of RNA-seq of different development stages was profiled and good repetition among three biological replicates in both CHA treated materials and control were obtained, which produced 139.7, 107.3, 151.1, 114.7 million raw reads for the control and 194.2, 147.6, 251.6 and 149.4 million raw reads for the CHA treated materials at YB stage (stage 1–5), SA, MA and LA stage, respectively. These raw data were filtered to get clean reads respectively, and then average 85.59% clean reads perfectly matched to the reference genome [28] (Additional file 2).

**Fig. 5:**
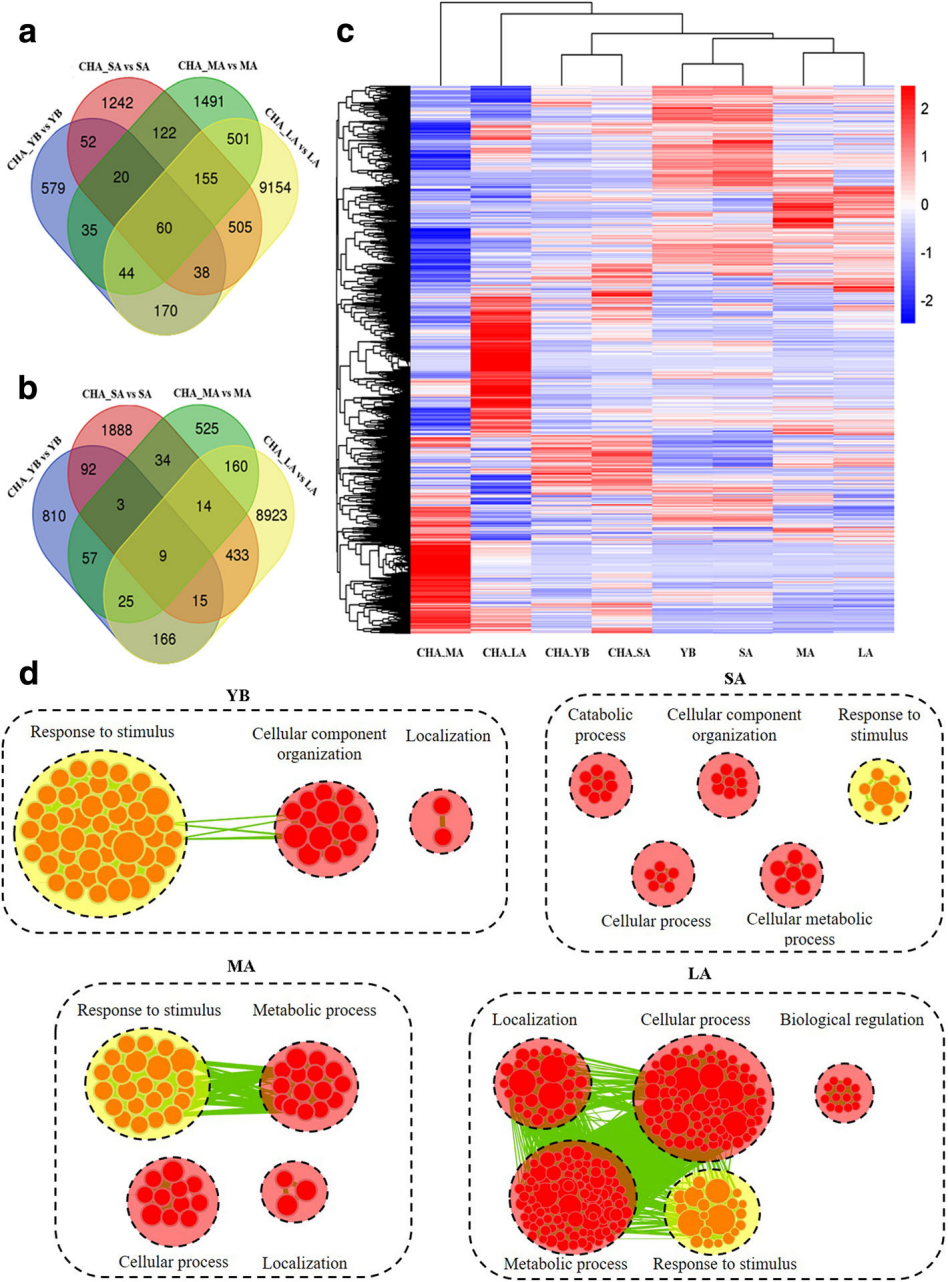
The heatmap for the DEGs between CHA treated materials and control and biological process analysis of down-regulated DEGs. **a**, Up-regulated genes. **b**, Down-regulated genes. **c**, The heatmap analysis of all DEGs at different stage. **d**, The yellow and red circles indicate the common and different biological processes among the four stages

The differential gene expression analysis was conducted, and a total of 998, 2 194, 2 428 and 10 627 up-regulated differentially expressed genes (DEGs) and 1 177, 2 488, 827 and 9 745 down-regulated DEGs (*P* < 0.05) were identified at YB, SA, MA and LA stage, respectively (Fig. 5a and b). The number of up- and down-regulated DEGs shared at all stages was only 60 and 9 respectively, while large number of DEGs in these adjoining stages were different. The hierarchical cluster analysis of all DEGs was performed to show the global gene expression pattern at different stages (Fig. 5c). As a result, CHA_YB and CHA_SA, YB and SA, MA and LA stage were located in the same cluster respectively, while CHA_MA and CHA_LA were far away from MA and LA respectively. Moreover, the differentially expression levels of several selected DEGs for further discussion were showed in Additional file 3. For example, two copies of *CSR1*, the target gene of SX-1, were down-regulated at YB stage.

As TFs usually play an important role in regulating at early stage [29], 68 up- and 144 down-regulated TFs were identified at YB stage (Additional file 4), and the top three TF families (ranked by DEG numbers) were “ERF” (41 DEGs), “MYB” (34 DEGs) and “NAC” (23 DEGs). Previous study had proposed a putative regulatory network model for Arabidopsis anther development, and *DYT1* (a putative bHLH TF) played the key role in the model, and *NAC025*, *MYB80* and *WRKY33* were the downstream genes of *DYT1* [30, 31]. *DYT1* didn’t show differentially expression at this stage (Additional file 3), while two copies of *NAC025*, four copies of *MYB80*, and six copies of *WRKY33* were significantly down-regulated at YB stage (Additional file 4). In Arabidopsis, *MYB80* (formerly named as *MYB103*) was restrictedly expressed at the tapetum of developing anthers and trichomes, and its down-regulation leaded to abnormal tapetum and pollen development [32, 33].

The up- and down-regulated DEGs at different stages between control and CHA treated materials were assigned for KEGG pathway analysis (*P* < 0.05). The results revealed that “flavonoid biosynthesis” and “protein processing in ER” were enriched at YB stage, “Phenylpropanoid biosynthesis” and “Galactose metabolism” were enriched at SA stage (Additional file 5), as several genes in these pathways were significantly differentially expressed, such as *Hsp70*, *CCOAMT*, *TT4* and *UGT72E3*. In Arabidopsis, *UGT72E1*- *E3* encode glycosyltransferases that glucosylated phenylpropanoids in vitro [34], and *UGT72E3* was predicted as the target of *bra-miR9556b-5p* in the present study. For MA stage, the down-regulated DEGs were enriched in “Protein export”, “Pyruvate metabolism” and “Fatty acid elongation” (Additional file 5). Among these genes, *IPT*s and *BRI1* were identified. *IPT*s mediated the rate-limiting step of cytokinin biosynthesis [35], and were the predicted target of *sample_miRNA_520* in the present study. At the last stage, many pathways related to amino acid metabolism were identified, including valine, leucine and isoleucine degradation, lysine degradation, alanine, aspartate and glutamate metabolism, etc. (Additional file 5). In the meanwhile, “Fatty acid degradation” and “Steroid biosynthesis” were enriched. Some important genes in these pathways were up- or down-regulated, for example, *LACS*s, *AIM* and *FK*. In Arabidopsis embryogenesis, *FK* was a sterol C-14 reductase, which was required for organized cell division and expansion [36], and was the predicted target of *sample_miRNA_201* in the present study. More importantly, the down-regulated DEGs at these stages shared one same pathway, protein processing in ER, which might imply the male sterility induced by SX-1 largely because of the disordered protein processing.

The down-regulated DEGs between CHA treated materials and control were further enriched by Cytoscape EnrichmentMap. As shown in Fig. 5d, YB, SA, MA and LA stage generated 64, 35, 52 and 251 nodes, respectively. These nodes were classified into different categories. Interestingly, the common terms among the four stages was “Response to stimulus”, which might largely due to the continued effect by SX-1 from early stage. Very importantly, some down-regulated DEGs at different stages were enriched in different categories, which might because of the continuous effect of SX-1 at different anther development stages. For instance, “Cellular component organization” was only enriched at YB and SA stage and “Catabolic process” was only enriched at SA stage. What’s more, “Localization”, “Cellular process”, “Cellular metabolic process” and so on were also clustered.

### Differentially expressed (DE) miRNAs between the CHA treated materials and control

**Fig. 6:**
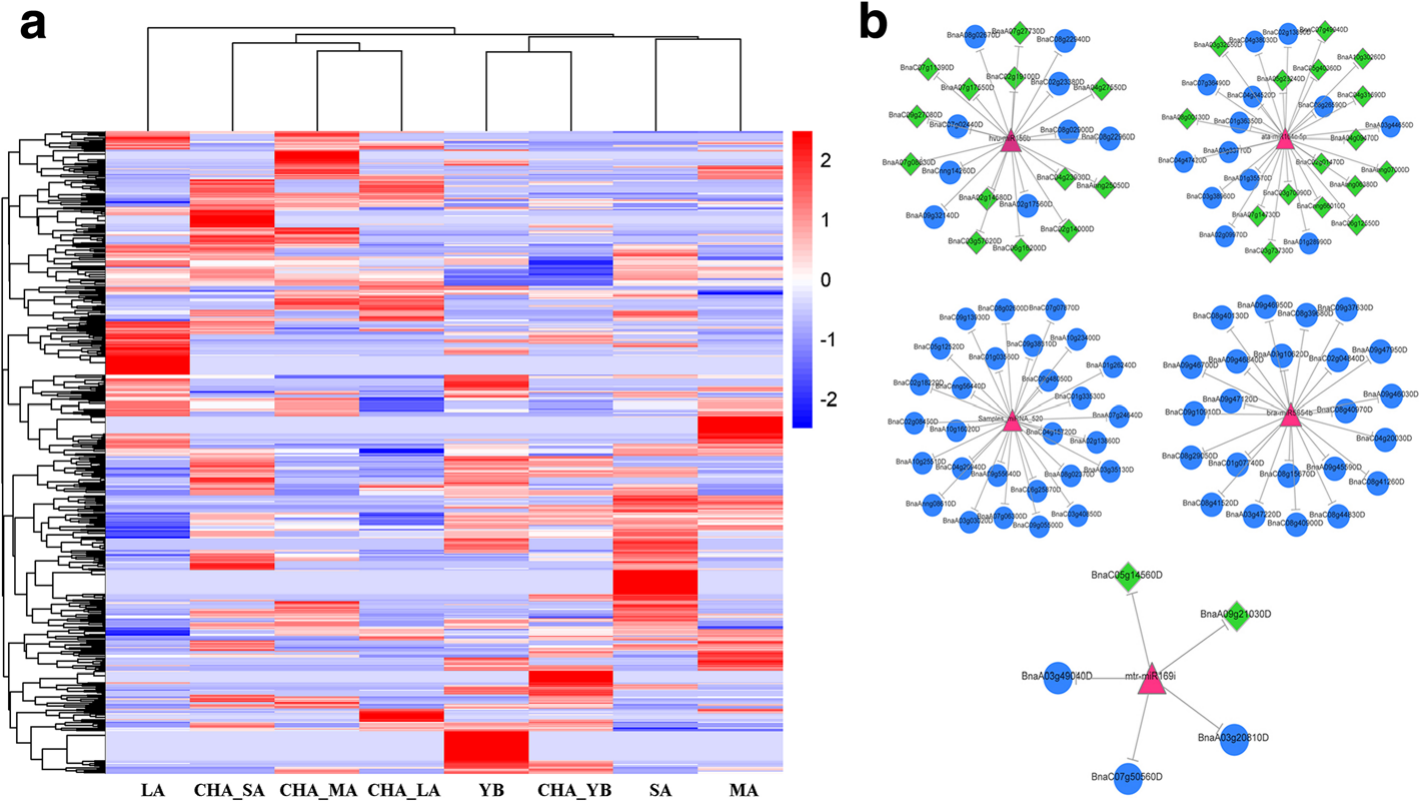
The heatmap analysis of miRNA expression between CHA treated materials and control and miRNA-mRNA regulatory network analysis in *B. napus*. **a**, The heatmap of miRNAs expression. **b**, miRNA-mRNA regulatory network analysis. Pink triangles, miRNA. Blue circles, functional genes. Green diamonds, TFs

A total of 125.9, 79.3, 97.9, 114.4 million raw reads and 86.3, 72.1, 73.7, 51.9 million raw reads were obtained at YB, SA, MA and LA stage in control and CHA treated materials, respectively. The length distribution of the small RNAs was analyzed and the 21–24 nt small RNAs were with the majority (the 24 nt small RNA was the most dominant) (Additional file 6). A total of 115 known miRNAs and 557 novel miRNAs were identified (Additional file 7). The hierarchical cluster analysis of all identified miRNAs was performed to show the different expression pattern at different stages (Fig. 6a). As a result, CHA_SA, CHA_MA and CHA_LA, YB and CHA_YB, SA and MA were located in the same cluster respectively, while LA stage was alone. Among the 672 miRNAs, 9, 14, 22 and 6 DE-miRNAs at YB, SA, MA and LA stage were identified, respectively (Additional file 8). Interestingly, these stages didn’t share even one common DE-miRNA, which suggested that miRNAs regulations induced by SX-1 at different anther development stages were obviously not alike.

Among the total 51 DE-miRNAs, 80, 101, 156 and 57 targets were identified for 7, 12, 20 and 5 DE-miRNAs at YB, SA, MA and LA stage, respectively (Additional file 9). Some miRNAs might be the important regulators, for example, *hvu-miR156b*, *bra-miR5654b* and one novel miRNA *Samples_miRNA_520* targeted more than 20 genes, *bra-miR9556-5p* and *mtr-miR169i* target 12 and 5 genes in the miRNA-mRNA network in *B. napus* (Fig. 6b). Interestingly, most targets of these DE-miRNAs were TFs. For instance, *hvu-miR156b* targeted *SPL*, *mtr-miR169i* targeted *WRKY* and *ata-miR164c-5p* targeted *NAC*, which suggested the timely miRNAs/TFs regulation modules might be essential for cellular reprogramming early in the response to SX-1. In addition, *bra-miR5654b* and *bra-miR158-5p* targeting PPR superfamily proteins and a novel miRNA *Samples_miRNA_520* targeting isopentenyl transferases (IPTs) were identified.

For the total 394 DE-miRNA/mRNA modules, 16 modules were inversely related, for example, *Samples_miRNA_500*, *bra-miR5654b*, *hvu-miR156b* and *bra-miR5654b* at YB, SA, MA and LA stage respectively, which targeted unknown protein, *PPR*, *SPL* and *PPR* respectively, whereas 20 modules were positively related (Additional file 10). Another 358 modules had no significant differential expression.

### The correlation analysis of transcript-to-protein between DEPs and DEGs

As *B. napus* is an allotetraploid plant, its genomes contain a large number of homologous sequences and repeats [37]. The 101 DEPs identified above, were corresponding encoded by 423 genes in total. Subsequently, correlation analysis between DEPs and DEGs was performed among different development stages. According to the expression in CHA treated materials compared to control, DEPs and DEGs can be divided into 4 groups: Group I, 23 (12 up-regulated and 11 down-regulated), 14 (10 up-regulated and 4 down-regulated), 53 (27 up-regulated and 26 down-regulated) DEGs showed the same expression trend with DEPs at SA, MA and LA stage respectively. For example, Hsp70, BIP2 and SHD, which are associated with protein processing in ER, SAM and GLN1.3, proteins related to amino acids biosynthesis, and KASI, a fatty acids biosynthesis protein. Group II, 17 at SA stage, 24 at MA stage and 46 at LA stage, these DEGs showed opposite expression trend with DEPs, such as TLP-3, a thaumatin-like protein 3 and CDC48, cell division cycle 48, which was functioned in ATPase activity and involved in response to cadmium ion; Group III, 369, 366 and 240 DEGs at SA, MA and LA stage respectively were not differentially expressed, for example, *GRF10* and *FBR12*, which were involved in protein phosphorylated amino acid binding; Group IV, 26 DEPs at SA stage, 34 DEPs at MA stage and 36 DEPs at LA stage didn’t show different expression, for instance, CAB1, a chlorophyll A/B binding protein and functioned in chlorophyll binding, and FIB, functioned in structural molecule activity and involved in photoinhibition. All the correlated DEGs and DEPs were displayed in Additional file 11. These results revealed that both independent and parallel correlations existed between the mRNA and protein expression profiles among different stages, which suggested there were a highly complex post-transcriptional regulatory network in the male sterility induced by SX-1.

### Validation of selected DEPs, DEGs, DE-miRNA by qRT-PCR and free amino acids, lipids and flavonoids between CHA treated and control materials

To validate the reliability of the RNA-seq and miRNA-seq, 15 DEGs and 8 DE-miRNAs potentially related to CIMS were selected for qRT-PCR (Additional file 12). The expression patterns of 12 DEGs and 6 DE-miRNAs detected by qRT-PCR were basically consistent with the RNA-seq and miRNA-seq, these results indicated that the RNA-seq and miRNA-seq in the present study were reliable. What’s more, among 6 DEPs encoded by 8 DEGs selected above, only the relative expression levels of *BnaC04g45570D*, *BnaA04g21720D* and *BnaA05g22420D* were consisted with the proteomic results, and the low correlation largely agreed with transcript-to-protein analysis mentioned above.

The comparative proteomic and transcriptomic analysis showed the male sterility induced by SX-1 might involve in amino acid (AA), fatty acid (FA) and flavonoid biosynthesis. To confirm these findings, the contents of total free AA, FA and flavonoids were analyzed at YB, SA, MA and LA stage of both CHA treated and control materials (Additional file 13). As a result, total free AA at YB, MA and MA stages were significantly reduced after SX-1 treatment. Moreover, the concentration of BCAA at YB and MA stages were also largely reduced after SX-1 treatment. What’s more, total flavonoids (including quercetin, kaempferol and isorhamnetin) were obviously reduced at YB and MA stage, while was increased at SA. The total FAs was significantly reduced at MA stage, further analysis revealed that palmitic acid (C16:0) was slightly decreased at YB and largely decreased at MA and LA, and linolenic acid (C18:3) was also reduced at YB, MA and LA, except for a slightly increase at SA. These data indicated that, amino acid, fatty acid and flavonoid biosynthesis might be destroyed to a great extent by SX-1, especially at MA stage.

### Interaction analysis for candidate genes and the putative metabolic pathway

**Fig. 7:**
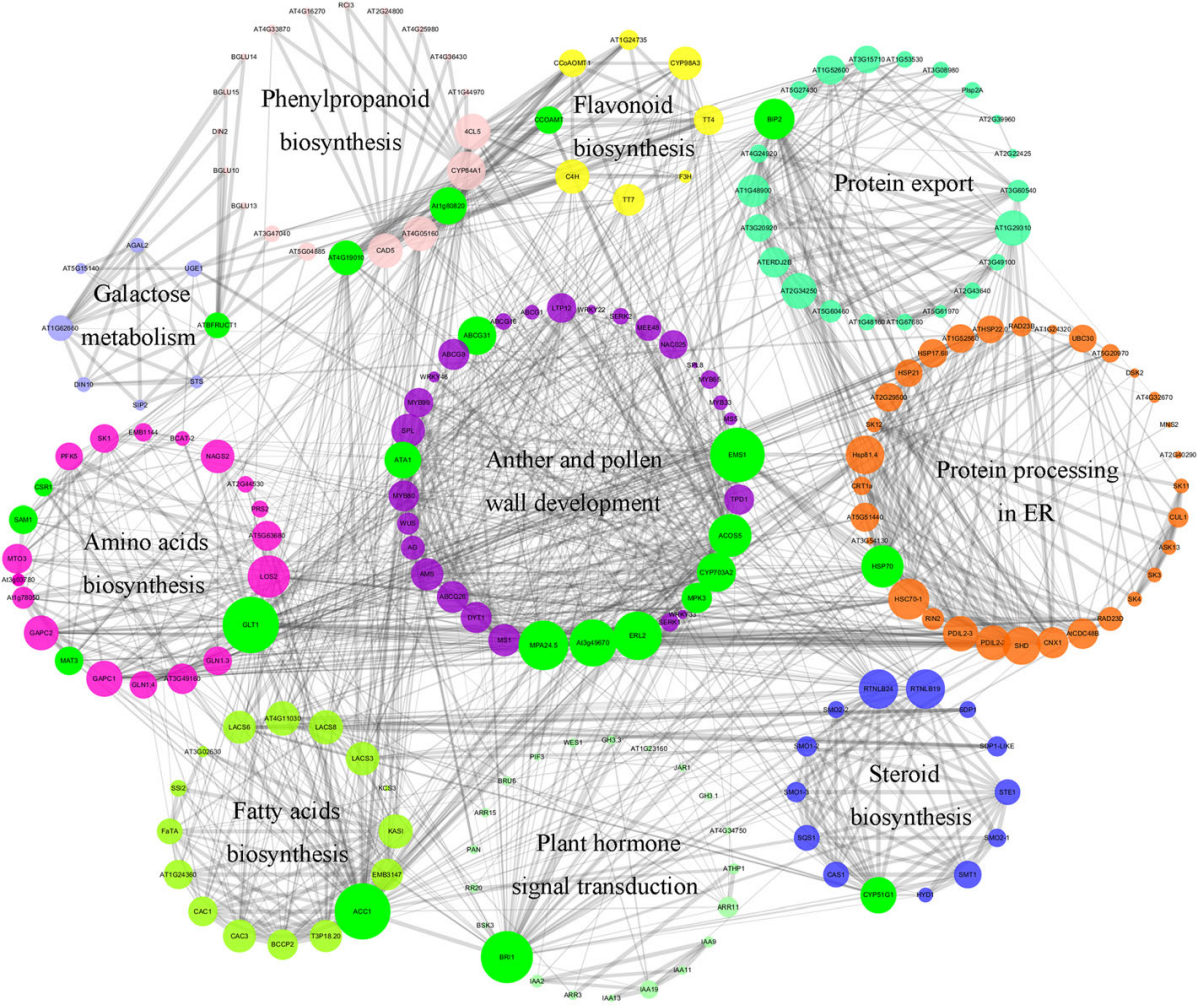
The interaction analysis of candidate genes. Node size are showed according to the interaction counts. Edge size are showed according to combined score. Gene associated with special type of pathway is shown using special color. Green node represents key gene in the interaction network

The results of proteome, transcriptome and miRNA-seq implied that male sterility induced by SX-1 should be controlled by a complex mechanism. To further elucidate this mechanism, we investigated the known and predicted interactions among the candidate genes corresponding to DEPs, DEGs, target genes of DE-miRNAs and genes reported to be involved in the development of anther or pollen wall based on *A. thaliana* orthologues (Additional file 14) by STRING 10.0, and then visualized by Cytoscape 3.6.1, and an interaction network associated with anther and pollen wall development were constructed (Fig. 7). It’s interesting that, some biologic processes enriched by KEGG analysis, including protein processing in ER, plant hormone signal transduction, protein export, galactose metabolism, biosynthesis of flavonoid, phenylpropanoid, amino acids, fatty acids, steroid, participated in the anther and pollen wall development through some pivotal proteins, such as EMS1, ACOS5, ERL2, ATA1 and ABCG31.

According to the interaction regulation network and the KEGG analysis (Additional file 5), an enormous metabolic pathway related to the male sterility induced by SX-1 at different stage was constructed (Additional file 15). Firstly, protein processing in ER and flavonoid biosynthesis were destroyed. Subsequently, phenylpropanoid biosynthesis, the upstream pathway of flavonoid biosynthesis was down-regulated at SA stage, might be for the tight interaction between 4CL5, CYP84A1, At1g80820, At4g05160, CAD5 and At4g19010 in phenylpropanoid biosynthesis and all the flavonoid biosynthesis proteins showed in Fig. 7. Meanwhile, galactose metabolism was also found down-regulated. DIN10, reported to responding to the level of sugar in the cell, was exhibited slight link with AT1G24320, a six-hairpin glycosidases superfamily protein in protein processing in ER. What’s more, one up-regulated *bra-miR9556-5p* were identified located at phenylpropanoid biosynthesis pathway by repressing the expression of *UGT72E3*. Afterwards, at MA stage, fatty acid elongation was affected, and the LACS3, LACS6, LACS8 showed strong interaction with AT4G19010, AT4G05160 and 4CL5 in phenylpropanoid biosynthesis, and CYP98A3, C4H and TT7 in flavonoid biosynthesis. As well as the protein export and plant hormone signal transduction displayed strong interaction with other pathway by some important proteins, such as BIP2, At1g29310, At2g34250, ATERDJ2B, BRI1 and BRU6. Interestingly, one novel miRNA, *samples_miRNA_520* targeting IPTs supposedly, which were of importance in zeatin biosynthesis, was significant up-regulated at MA stage. Finally, amino acids, fatty acids and steroid biosynthesis at the LA stage were down-regulated, which were the upstream of pyruvate metabolism, fatty acid elongation and brassinosteroid biosynthesis respectively, while these pathways were all down-regulated at MA stage. Meanwhile, the fatty acid degradation was up-regulated. Additionally, one novel miRNA, *samples_miRNA_201* was supposed to target FK exhibiting up-regulated at steroid biosynthesis.

### A potential regulation model for male sterility induced by SX-1

**Fig. 8:**
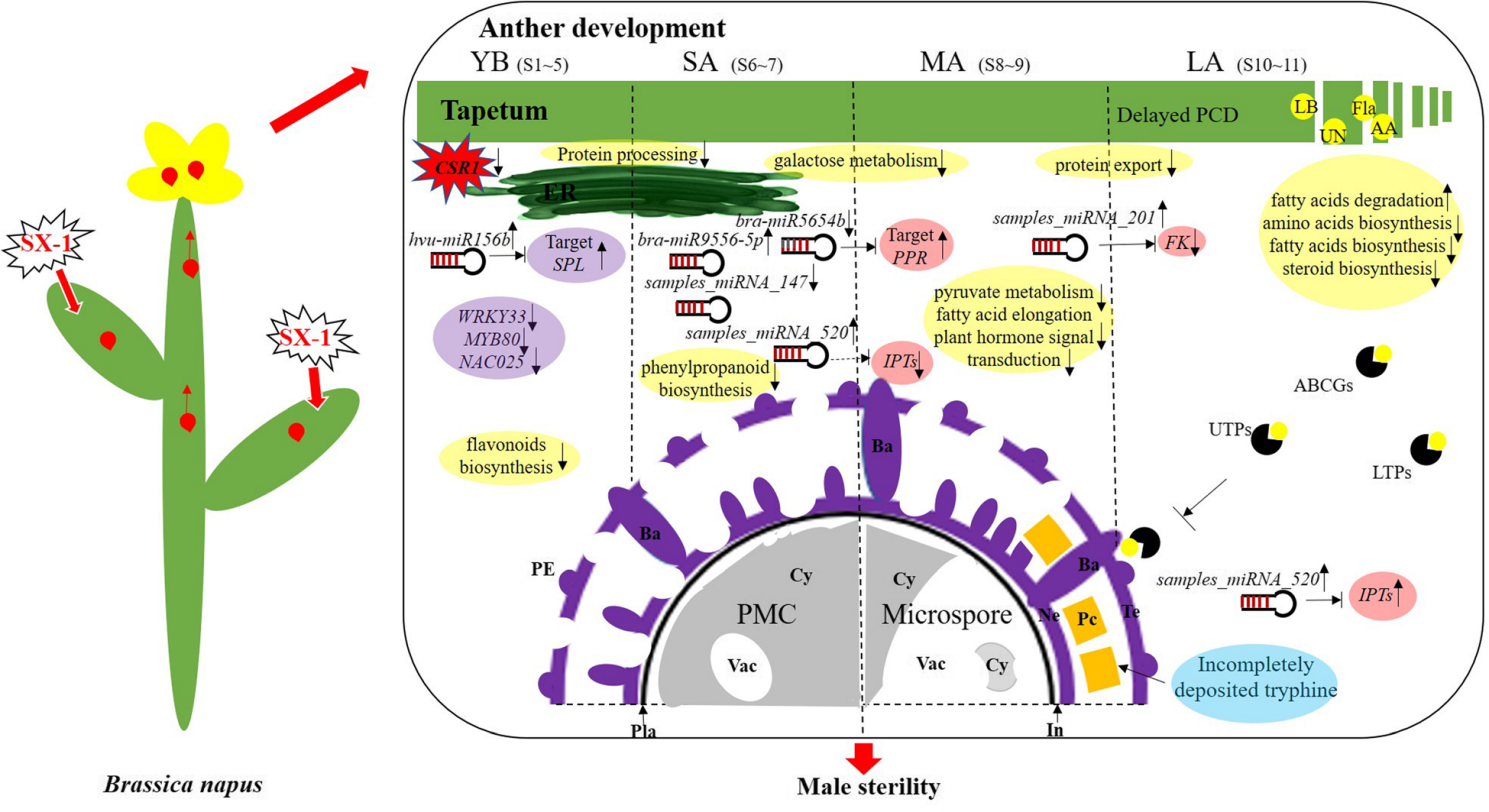
A proposed model for male sterility induced by SX-1 in *B. napus*. ER: endoplasmic reticulum, PE: primexine, Pla: plasma membrane, Te: tectum, Ba: bacula, In: intine, Ne: nexine, Pc: pollen coat (tryphine), Cy: cytoplasm, Vac: vacuole, LBs: lipid bodies, AA: amino acid, Fla.: flavonoids, UN: unknown nutrients, UTPs: unknown transport proteins, PMC: pollen mother cell. Up arrow, up-regulation. Down arrow, down-regulation

According to the results mentioned above, we proposed a possible regulation model for male sterility induced by SX-1 in *B. napus* (Fig. 8). After spraying on leaves, SX-1 was mainly absorbed by mesophyll, and then polar-transported to the anthers largely through the phloem. Firstly, *CSR1*, the target gene of SX-1 was down-regulated. At the same time, the accumulation of SX-1 in the anthers might affect the expression of several important TFs, such as *SPL, MYB80* and *NAC025*, partially through the regulation of TFs/miRNAs modules, for example, *miR156/SPL* at YB stage, when the protein processing in ER and flavonoid biosynthesis were destroyed. Secondly, probably because of the disordered flavonoid biosynthesis ahead, its upstream pathway, phenylpropanoid biosynthesis which associated with *bra-miR9556-5p* were down-regulated at SA stage, as well as galactose metabolism. Thirdly, besides the galactose metabolism, due to the early cellular reprogramming in the response to SX-1, plant hormone signal transduction, pyruvate metabolism and fatty acid elongation were all down-regulated. Moreover, *samples_miRNA_520*, predictively targeting *IPT*s was identified up-regulated. In the meanwhile, *bra-miR5654b/PPR* modules were disordered. Then the delayed PCD process in tapetum during mitosis was happened, and the tapetum cells stacked together subsequently at LA stage. At the same time, some upstream pathways of the down-regulated ones at MA stage, such as steroid, amino acids and fatty acids biosynthesis were also imbalanced. Meanwhile, the function of some transport proteins, such as LTPs, ABCGs were impeded. Finally, the tapetum couldn’t timely provide young microspores nutrients, such as flavonoids, amino acids and lipids, resulting in the defective in tryphine of pollen wall and completely crinkled pollen grains with little cytoplasm and abnormal high content of vacuole, and male sterility at last.

## Discussion

The first CHA was reported in 1950 [38], more and more CHAs had been developed in the following decades for the production of hybrid seeds [9, 39]. Previous study showed that, genes involved in cellular transport, lipid and carbohydrate metabolism were differentially expressed in MES induced male sterility [11]. TM resulted in BCAA starvation by anther-specific acetolactate synthase (*ALS*) inhibition and autophagic cell death in anthers [9]. The oxidative stress and overexpression of type II metacaspase in SQ-1 treated wheat would trigger premature tapetal apoptosis, and then result in pollen abortion [12, 13]. Here, we found the new ALS inhibitor SX-1 can be used as an effective CHA to induce male sterility in *B. napus*. In the present study, a low correlation level was found between the transcriptome and proteome. While this was normal for it has also been reported in other species, such as soybean [40], cabbage [41] and citrus [42]. This phenomenon might be resulted from that transcripts and proteins represent two different biological levels, and the modification and degradation of protein might affect the proteomic data [40, 41].

### SX-1 disturbed protein processing in ER and flavonoids biosynthesis at early stage

In previous study, during development of male sterile pepper anther, amino acid synthesis was significantly changed accompanying abnormal tapetum maturity [43]. The present results revealed that not only the content of BCAA, but the total free AAs were also significantly reduced at YB stage, which might largely due to the down-regulation of *CSR1*, the target gene of SX-1. At this stage, protein processing in ER and flavonoid biosynthesis were enriched. As we all know, almost all the proteins in the cells were processed in ER, so the defective in protein processing and the decreased total free AAs would repress the biosynthesis of proteins with different function in various pathways. Interestingly, Hsp70 and BIP2 were down-regulated, and had strong interaction with down-stream pathways, such as plant hormone signal transduction, amino acids and fatty acids biosynthesis and protein export (Fig. 7 and Additional file 15). Moreover, CSR1 also shows interaction with Hsp70. Hsp70 is a kind of molecular chaperone and plays numerous crucial roles in protein folding [44] and BiP is a molecular chaperone of Hsp70 family and plays important roles in protein translocation, folding and quality control in the ER [45]. In Arabidopsis, the *bip1 bip2 bip3* triple mutation was lethal in pollen [46]. What’s more, protein processing in ER was the only common down-regulated pathway enriched at the four identified stages. Hence, we could presume that, the decreased content of total free AAs and the down-regulation of some key genes in ER cannot ensure ER homeostasis in cells, for the increased protein secretion activity is required to satisfy the protein processing demand at the rapidly growing stage of anther.

Flavonoids played crucial roles as UV protectants, pigments, attractants for pollinators, auxin transport and regulators of fertility [47, 48], and are the major components of tryphine [22]. As we mentioned above, TEM observation showed the tryphine deposited incompletely after SX-1 treatment and several flavonoids biosynthesis genes were down-regulated at YB stage, such as *CCOAMT, TT4* and *C4H*. In addition, all of these genes linked tightly with some important genes located at phenylpropanoid, amino acids, fatty acids and steroids biosynthesis and plant hormone signal transduction pathways, for instance, *4CL5*, *SAM1*, *LACS3*, *RTNLB19*, and *BRI1* (Fig. 7 and Additional file 15). In Arabidopsis, *miR156*/*SPL* are known to have important roles in regulating phase change and flowering, and 10 of the total 16 *SPL*s are targets of *miR156* [49, 50]. In the present study, *hvu-miR156b* was up-regulated at YB stage. *SPL8* affect reproductive development through the genes involved in Gibberellic acid biosynthesis, and the *spl8* mutant showed reduced fertility by generating abnormally developed microsporangia [51], while constitutively overexpressed *miR156* in the *spl8* mutant background would result in fully sterile plants [52]. What’s more, a previous study indicated *miR156*/*SPL* module negatively regulate flavonoids biosynthesis in Arabidopsis [53]. The total flavonoids contents at YB stage was significantly decreased. Therefore, we could make a speculation that the disordered flavonoid biosynthesis directly affected by SX-1 at early stage will cause defective of other related pathways, and finally results male sterility.

### SX-1 influenced multiple biosynthesis and metabolism during anther development stage

In previous study, the long chain lipids were important components of the plant cuticle that established the boundary surface of aerial organs, and these lipids were detected in the tryphine, where they played a critical role in appropriate communication between pollen and stigma [23, 54]. The mutations of two long-chain acyl-CoA synthetases in Arabidopsis, *lacs1* and *lacs4* clearly showed a cooperation between LACS1 and LACS4 in the process of pollen ripening, which have been respected to produce tryphine lipids [23]. CER6 is critical for the biosynthesis of very long-chain fatty acids, and the mutation of CER6 in *Arabidopsis* resulted in conditional male sterility [54]. *FLP1* has the similar sequence with proteins that involved in wax synthesis, and the previous study showed that FLP1 protein is likely to play an important role in the biosynthesis of the components of tryphine and sporopollenin of exine [55], and it has been demonstrated that sporopollenin is consist of fatty-acid derivatives and polymerized phenols [56]. Moreover, a mutant deficient in steryl glycoside biosynthesis, *ugt80A2 ugt80B1*, exhibited immature tryphine, which indicted steryl glycosides were improtant for pollen fitness by supporting pollen coat maturation [57]. In the present results, the tapetosomes and elaioplasts in tapetum cells degraded in advance, then the PCD process of the tapetal cells were delayed, and ultimately stacked together (Fig. 2y and z), which resulting in the aberrant of pollen, with tryphine deposited incompletely and cytoplasm of the microspores became highly concentrated and abnormally vacuolated. What’s more, we enriched the steroid and fatty acids biosynthesis on down-regulated genes and fatty acid degradation on up-regulated genes during anther development by KEGG analysis (Additional file 5). In accordance with these results, the present physiologic analysis showed that, total fatty acids at MA stage were significantly decreased in CHA materials. What’s more, C16:0 and C18:3 were decreased in male sterile pollen coat extract (PCE) [4], which was largely consist with our results.

Some key genes associated with biosynthesis and metabolism of amino acids, fatty acids and other metabolites linked to anther and pollen wall development through *EMS1*, *ACOS5*, *CYP703A2*, *MPK3*, *ATA1*, etc. (Fig. 7 and Additional file 15)*. ATA1* specific expressed in tapetum with a conserved role in tapetum development, and significantly down-regulated in several male mutants, such as *dyt1*, *ems1* and *ams1* [18, 58, 59]. Here, we found *ATA1* down-regulated after SX-1 treatment, which directly interacted with several anther and pollen wall development and amino acid biosynthesis genes, a phenylpropanoid biosynthesis gene *CAD5* and a lipid metabolism related gene *KASI*. As lipids were the main component of tryphine in pollen wall, genes related to fatty acids biosynthesis and lipid transport were essential for tryphine formation [17, 60]. It’s interesting that, our results indicated that *KASI* and *LACS6,* which were crucial for lipid metabolism, and lipid transfer protein (*LTP*)12, were down-regulated by SX-1 (Additional files 1 and 3). In addition, *ACOS5*, a fatty-acyl-CoA synthetase gene which preferentially expressed in the tapetum, and the *acos5* mutant couldn’t produce pollen in mature anthers or apparently lacked sporopollenin or exine in pollen wall formation [61]. In the present study, two copies of *ACOS5* was slightly down-regulated at SA stage and formed tightly interaction with *CYP703A2*. In the previous study, *MS2* and *CYP703A2* tightly co-expressed with *ACOS5*, and despite the similar phenotypes of loss-of-function between these 3 genes, *acos5* was completely male sterility while *ms2* and *dex2* (*CYP703A2* knockout mutants) showed low levels of fertility. It has been proposed that *ACOS5* might act more centrally in the sporopollenin biosynthetic pathway than *CYP703A2* and *MS2* [61, 62]. As showed in the present interaction network, *ACOS5* showed more interaction counts than *CYP703A2,* which might be consisted with foregoing proposal. In addition, two ATP binding cassette transporters, *ABCG9* and *ABCG31*, which were involved in pollen coat deposition and necessary for the transfer of steryl glycosides from tapetum to pollen surface [57], were down-regulated at different stages after SX-1 treatment (Additional file 3), which implied the defective transfer of steryl glycosides from tapetum to pollen wall were happened, and then cause the incompletely deposition in tryphine.

In plants, miRNAs predominantly regulate their targets by the direct cleavage of the target mRNAs [63, 64]. Therefore, most of the miRNA/mRNA modules are inversely related. In this study, the expressions of most miRNAs were not greatly inversely related with the mRNAs of their target genes, for only 16 modules were inversely related among the total 394 DE-miRNA/mRNA modules. It seemed that miRNAs had a limited role in the regulation of male sterility induced by SX-1, which was similar with the miRNAs in the regulation of taxol biosynthesis reported before [65]**.** Some previous studies show that miRNAs play important roles in anther development [51, 52, 66]. For example, miRNA159 represses GAMYB-like genes which encode R2R3 MYB domain transcription factors, and result in defective anther or male sterility in *Arabidopsis* [66]. In the present study, bra-miR9556-5p was up-regulated after CHA treated, while its target UGT72E3, which was important in phenylpropanoid biosynthesis (Additional file 15) was down-regulated. What’s more, two novel miRNAs, Samples_miRNA_520 and Samples_miRNA_201, which target IPTs in zeatin biosynthesis pathway and FK in steroid biosynthesis pathway respectively, were all up-regulated. These results indicated that, miRNAs might through some indirect ways to cause male sterility in SX-1 treated plants, and these need further study.

According to the present proteomic and transcriptome results, several genes which were known to be involved in early anther development in these interaction network didn’t show significantly different expression during early stages (Additional file 3). For example, *BAM1* and *BAM2*, which played an early role in promoting somatic cell fates and a subsequent function in the pollen mother cells [67], *ERL2*, which played important roles in anther cell differentiation and normal anther lobe formation [68], and *EMS1*, which controlled the patterning and cell fate in anther development [69]. Interestingly, these results were largely corresponding with the ultrastructural cytological analysis, for there were no difference observed between CHA treated materials and control anther before stage 8 by both SEM and TEM observation.

Taken together, candidate genes in the network could be classified into three types in general. For type I, genes related to early anther development, such as *BAM1*, *EMS1* and *ERL2* were not affect by SX-1 at early stages while showed differentially expressed at later stage (Additional file 3), which were largely in accordance with the phenomenon that the early structure of anther was developed normally in CHA materials. Even though these genes didn’t differentially express earlier, they participated in the male sterility induced by SX-1 by strongly interacting with some crucial genes at other pathways. For type II, genes involved in amino acids, flavonoids, fatty acids and steroid biosynthesis, metabolism, or transfer in anther, such as *MAT3*, *GLN1.3*, *CCOAMT*, *KASI, ACOS5, ABCG9*, *LTP12*, and so on, showed significantly different expression after SX-1 treatment (Additional files 1 and 3). Especially *CCOAMT*, related to flavonoid biosynthesis, was largely down-regulated at YB stage. For type III, several transcript factors were obviously down-regulated from early stage, including *WRKY33*, *NAC025*, *MYB80*, etc. (Additional file 4). Some of these genes were identified as the target genes of DE-miRNAs, which indicated the regulation of anther and pollen development by miRNAs were severely affected by SX-1. In summary, genes in type II and type III predicted to be of great importance in male sterility induced by SX-1, while the detail interaction patterns between these genes and function identification in *B. napus* need further study.

### SX-1 affects Rf-like *PPR* genes expression which related to CMS

In higher plants, the CMS phenotype could be recovered by dominant nuclear genes, named restorer of fertility (*Rf*) genes, which could specifically reduce the accumulation of CMS-associated aberrant chimeric gene in the mitochondria [70]. Most of the *Rf* genes encoded PPR proteins [71]. To date, two *PPR* genes were demonstrated as the restoration genes for CMS in *B. napus*, *Rfp* for *pol* CMS [72] and *Rfn* for *nap* CMS [71], and 53 Rf-like *PPR* genes were indentified in *B. napus* (unpublished data), using the Rf-like *PPR* genes in Arabidopsis *(AtRFL1 ~ 26*) [73] as query sequences to blast in the *B. napus* genome one by one. We found that four of these Rf-like *PPR* genes in *B. napus* (*BnaA09g46690D*, *BnaA09g46900D*, *BnaA09g47120D* and *BnaA10g07840D*) were significantly down-regulated after SX-1 treatment, especially at SA stage (Additional file 3), which were largely consisted with our analysis mentioned above, for several *PPR* genes were predicted as the target genes of the up-regulated DE-miRNAs, *bra-miR5654b* and *aly-miR158a-3p*. The *BnaA09g46690D* and *BnaA09g47120D* were the restoration genes for *pol* CMS and *nap* CMS respectively [71, 72]. Hence, we could make an assumption that, Rf-like *PPR* genes which were important for CMS might also play key roles in CIMS, and probably through the regulation of miRNAs.

## Conclusion

In this study, the proteomic, transcriptomic and miRNAs, combined with morphological and physiological analysis were conducted. Earlier degeneration of the tapetosomes and elaioplasts, aberrantly stacking in tapetal cells, deletion of organelles and cytoskeleton in pollen grains, and incompletely deposition in tryphine of pollen wall were observed in CHA treated *B. napus* through SEM and TEM analysis. It was revealed that the deficiencies in protein processing in ER and flavonoids biosynthesis were occurred at early stage in the CHA treated materials. Subsequently, plant hormone signal transduction, biosynthesis of amino acids, fatty acids and steroid in anther at later stages were identified down-regulated after SX-1 treatment. Moreover, some TFs were also indentified to down-regulated at early stage, which suggested the early regulation in anther and pollen wall development were disordered in CHA treated *B. napus*. Additionally, some important miRNAs were also identified and some of the predicted target genes of miRNAs were Rf-like genes.

Accordingly, an interaction network of candidate genes and the putative metabolism pathway were conducted based on the multi-omics integrative analysis provided new insights into the male sterility induced by SX-1 in *B. napus*. This study lays a foundation for revealing the mechanism of male sterility induced by CHA. Besides, these results can provide more potential targets for creating new CHAs to induce male sterility in *B. napus* or other crops.

## Methods

### Plant materials and SX-1 application

Winter-type *B. napus* lines 7792–95/772/772 were cultivated in the experiment field of Rapeseed Hybrid Center of Shaanxi province in 2010–2017 (Dali and Yangling, Shaanxi province, China) and Huazhong Agriculture University in 2015–2018 (Wuhan, Hubei province, China). The materials were sprayed with 6 mg/L SX-1 on leaves during the budding stage with the longest bud over 2 mm, and the control were treated with distilled water. The dose was 2.5 mL per plant.

### Observation of SEM and TEM

Anthers at different development stages were vacuum-infiltrated and fixed with 2.5% (*w*/*v*) glutaraldehyde in 0.1 M phosphate buffer (pH 7.4). The following procedures were followed as previous study [74]. For scanning electron microscope (SEM) analysis, anther samples were dehydrated through a graded series of ethanol (70, 85, 90, 95%, 2 × 100%), deal with isoamyl acetate and dried with a critical point dryer (HITACHI HCP-2), mounted on SEM stubs using silver paint and then images were acquired using a S-3000 N electron scanning microscope (Hitachi, Japan) [74]. For transmission electron microscope (TEM) observation, anther samples were post-fixed with 1% osmium tetroxide solution after phosphate buffer rinsing, then washed with ultrapure water, dehydrated through a graded series of acetone (20, 50, 70, 90% and 3 × 100% *v*/v). After infiltration through a graded acetone/Epon/Spurr’s epoxy resin series, samples were embedded in 100% (w/v) Spurr’s epoxy and polymerized at 60 °C for 24 h. Ultrathin sections were prepared using a Diatome diamond knife on an UC6 Ultratome (Leica, Germany) onto copper grids and stained with uranyl acetate and lead citrate. Images were viewed and collected under an H-7650 transmission electron microscope (Hitachi, Japan) [74].

### Proteomic analysis

After treated with SX-1 or distilled water, flower buds were collected at the four different anther development stages (named as SA, MA, LA and LA2, which represented the anther development stage 6–7, stage 8–9, stage 10–11 and stage 12–13 respectively, according to the division of anther development stage in Arabidopsis [75]) of CHA treated and control plants. The anthers were taken out from the buds on ice and quickly frozen by liquid nitrogen, and kept in − 80 °C.

The preparation of total protein was performed according to our previous report [76] with little modifications. Briefly, ~ 0.5 g of anther samples was homogenized to powder in liquid nitrogen and then added precooled 10% TCA/0.07% dithiothreitol (DTT) in acetone and incubated at − 20 °C overnight. Then centrifugated at 20 000 g for 30 min at 4 °C, and the pellets were washed three times with 1.5 mL of precooled acetone containing 0.07% DTT and then centrifuged again. The sample pellets were then solubilized in the lysis buffer. Then the solution was incubated at 25 °C for 1 h and then centrifuged at 12 000 g for 20 min, and the supernatants were collected into new tubes and stored at − 80 °C.

The 2-DE analysis was also largely according to Gan et al [76] with minor modifications. Briefly, the 24 cm length and pH gradient 4 ~ 7 of IPG strips were rehydrated with 450 μL of rehydration solution containing 0.5 mg of protein for 14 h at 20 °C in an IPG Box. After IEF, the strips were then equilibrated for 15 min. Then the second dimension separation was conducted on 12.5% SDS-PAGE gel. Finally, the proteins on gels were visualized by the colloidal Coomassie Brilliant blue G-250.

The DEPs analysis was also conducted according to our previous report [76]. Briefly, the gel matching and spot detection were analysis with PDquest version 8.01 software. Then the spots were all normalized to the total density. Finally, the least significant difference performed > 95% (*p* < 0.05) and the spots which changed in abundance over 2-fold were selected for protein identification. The interested spots were manually cut from gels and washed three times with Milli-Q water. The protein digestion and MALDI-TOF-MS-MS analysis were absolutely according to Gan et al [76].

### Transcriptomic analysis

On the purpose to identify the key genes responsible at early stage, shorter than 1.5 mm young buds (YB stage, anther development stage 1–5) were selected. What’s more, SA, MA and LA stage were also selected for transcriptomic analysis. The extraction of total RNA, assembly and mapping of the clean reads, and transcriptome annotation were conducted according to our previous report [77] with minor modification.

Briefly, 0.1 g samples were used to extract the total RNA by TRIzol reagent (Invitrogen, Carlsbad, CA, USA) according to the manufacturer’s instructions. The cDNA sequencing libraries were constructed by TruSeq™ RNA Sample Preparation Kit (Illumina, San Diego, CA, USA). Firstly, polyA mRNA was purified from the total RNA. Secondly, the purified poly-A mRNA was fragmented into 200 ~ 700 bp pieces. Then these fragments were used as templates for the first-strand cDNA synthesis, and then used RNase H and DNA polymerase I to synthesize the second-strand cDNA. Then these fragments were purified, A-tailed, end-repaired and ligated to index adapters (Illumina). To generate cDNA libraries, the ligated products were PCR-amplified and then using the HiSeq X Ten sequencing platform to conduct the sequencing. After sequencing, the raw reads were filtered by NGSQC toolkit (v2.2.3) to generate high quality clean reads. Then these clean reads were mapped to *B. napus* reference genome by Tophat2 (v2.0.13). Expected number of Fragments Per Kilobase of transcript sequence per Millions base pairs sequenced (FPKM) method was chose to calculate the expression levels of transcripts. All of the expressed transcripts with FPKM equal to or more than 1 were thought as expression. The DEGs between CHA treated plants and control at different stage were evaluated by DESeq2 software with an adjusted *p*-value < 0.05 and normalized fold change ≥2.

### DE-miRNA analysis

Firstly, the clean data (18–26 nt) was blasted in *B. napus* genome by Bowtie software; Secondly, the resulting data was used to identify miRNA by Mireap software (https://sourceforge.net/projects/mireap/), with the default parameters; Finally, the miRNAs in all samples were gathered together, and then the redundancy sequences were deleted. For miRNA annotation, the mature miRNA sequences were blasted in miRbase 21 database (http://www.mirbase.org/). To predict target genes of miRNAs, we used the Target Finder v1.6 software according to methods described as previous study [78].

### qRT-PCR

For DEGs, total RNA was reverse-transcribed using ReverTra Ace® qPCR-RT Master Mix with gDNA Remover (TOYOBO) according to the manufacturer’s protocol and *actin* was used as an internal reference [79]. For DE-miRNAs, we performed the stem-loop qRT-PCR. At first, total RNA was reverse-transcribed using a specific stem-loop primer by Goldenstar™ RT6 cDNA Synthersis Kit, and *U6* was selected as an internal control for data normalization [80]. The qRT-PCR experiments and the calculation were conducted according to our previous report [77]. All primer sequences were listed in Additional file 16.

### Analysis of free amino acids, lipids and flavonoids

Total free amino acids were extracted from ~ 100 mg of freeze-dried YB, SA, MA and LA samples of CHA treated materials and control respectively. The extraction process was followed by previous study [81]. Free amino acids were quantified on a Hitachi L-8800 amino acid analyzer, according to the manufacturer’s instructions [82].

Total flavonoids analysis was followed by previous study [83] with some modification: ~ 100 mg samples were circulation reflux with 10 mL of methyl alcohol and 25% HCl mixed liquor (the ratio was 4:1) for 30 min at 95 °C, then quickly cooled down. The extraction was filtrated with 0.45 μm Nylon Syringe Filter (ANPEL Laboratory Technologies, SCAA-104) before analysis. The determinations of total flavonoids content including kaempferol, isorhamnetin and quercetin.

To analysis the total fatty acids from YB, SA, MA and LA samples of CHA treated materials and control, we followed the method described in Zhang et al [84] with minor modification. Roughly as follows, firstly, ~ 60 mg of freeze-dried sample was weighed in glass tube, then 1.5 mL of 2.5% sulphuric acid in methanol, 400 μL toluene, and 200 μL of 2 mg/mL C17:0 in toluene (Nu-Chek Prep, Elysian, MN, USA) as an internal standard were added to each tube. Secondly, the tubes were capped and heated at 90 °C for 1 h in water bath. Thirdly, fatty acid methyl esters which was generated by the transesterifcation reaction were extracted by addition of 1 ml heptane and 1.8 ml H_2_O to each tube, and after fully mixed, the mixture were restored at room temperature for 12 h. Finally, the heptane layer was filtrated with 0.45 μm Nylon Syringe Filter (ANPEL Laboratory Technologies, SCAA-104) before analysis. The analysis of fatty acid composition was detected by gas chromatography using MIDI Sherlock® Microbial Identification System.

## Additional files

Additional file 1: The identification and relative expression level of DEPs. (XLSX 29 kb)

Additional file 2: Summary of output and mapping of the clean reads from the different samples. (XLSX 11 kb)

Additional file 3: The Log 2 ratio (CHA/control) of some selected DEGs in *B. napus*. (XLSX 11 kb)

Additional file 4: The differentially expressed TFs at YB stage. (XLSX 26 kb)

Additional file 5: Enriched KEGG pathway identified from up- and down-regulated genes. Pathways were considered enriched at *P* < 0.05. (XLSX 21 kb)

Additional file 6: Length distribution of small RNAs. (JPG 560 kb)

Additional file 7: All miRNAs identified by miRBase21. (XLSX 32 kb)

Additional file 8: DE-miRNA analysis at different stage. Inf indicate the miRNA only detected in CHA treated materials, #NAME? indicate the miRNA only detected in control. (XLSX 13 kb)

Additional file 9: The predicted target genes for DE-miRNAs. (XLSX 50 kb)

Additional file 10: DE-miRNA/mRNA modules of inversely and positively related. Inf indicate the miRNA only detected in CHA treated materials, #NAME? indicate the miRNA only detected in control. (XLSX 15 kb)

Additional file 11: The Log 2 ratio (CHA/control) of all the correlated DEGs and DEPs. r_SA, r_MA and r_LA result from RNA-seq at SA, MA and LA stage respectively. p_SA, p_MA and p_LA result from proteomic analysis respectively. (XLSX 37 kb)

Additional file 12: Validation for DEGs and DE-miRNAs by qRT-PCR. a, Result from RNA-seq and miRNA-seq. b, Result from qRT-PCR. The numbers are log_2_^X^-normalized ratio values. Red color represents higher gene expression levels. Green color corresponds to lower gene expression levels. The blocks without a numerical value indicate the gene expression was not detected by RNA-seq or miRNA-seq. (JPG 110 kb)

Additional file 13: Analysis of free amino acids, total flavonoids and fatty acids of the control and CHA materials. The values indicate means ± s.d., *n* = 3, *P < 0.05, **P < 0.01, by Student’s t test. (JPG 163 kb)

Additional file 14: The candidate genes used for interaction analysis. (XLSX 16 kb)

Additional file 15: The putative metabolic pathway for male sterility induced by SX-1. Red letters indicate down-regulation, and green letters indicate up-regulation. Gray dotted line means interaction between these two genes. Gray dotted arrow means there are many steps between the two metabolites. Gray two-way arrow means the same metabolite or pathway. Pathways located in the light blue background indicate the main events at the YB stage, light green for SA stage, light red for MA stage and light purple for LA stage respectively. (JPG 1163 kb)

Additional file 16: All Primers used in qRT-PCR and the reverse transcription of miRNAs. The sequence indicts the universal stem-loop are underlined. (XLSX 19 kb)

## Abbreviations

2-DE: Two-dimensional electrophoresis
*ALS*: Acetolactate synthase
BCAA: Branched-chain amino acid
CHA: Chemical hybridization agent
CIMS: Chemical induced male sterility
CMS: Cytoplasmic male sterility
DEGs: Differentially expressed genes
DE-miRNAs: Differentially expressed miRNAs
DEPs: Differentially expressed proteins
ER: Endoplasmic reticulum
GMS: Genic male sterility
IPTs: Isopentenyl transferases
*LTP*: Lipid transfer protein
MES: Monosulphuron ester sodium
PMCs: Pollen mother cells
TF: Transcript factor
TM: Tribenuron-methyl
