# Supplementary figures and images for: The initial deficiency of protein processing and flavonoids biosynthesis were the main mechanisms for the male sterility induced by SX-1 in Brassica napus
Source: BMC Genomics. 2018 Nov 7;19:806. doi: 10.1186/s12864-018-5203-y (PMC6223035; doi:10.1186/s12864-018-5203-y)

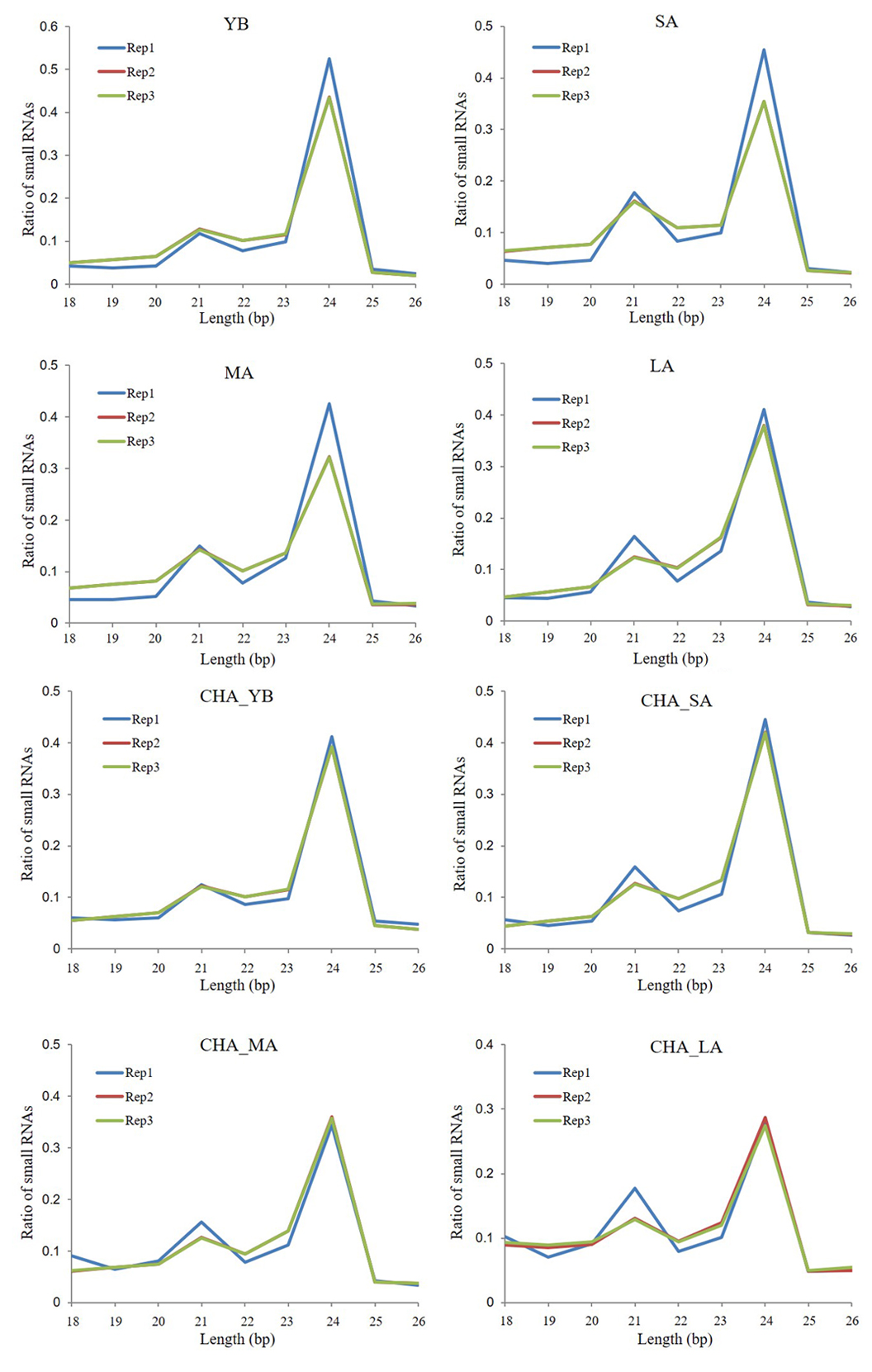

Supplement: Supplementary file 6 — Length distribution of small RNAs. (JPG 560 kb) [file 12864_2018_5203_MOESM6_ESM.jpg]

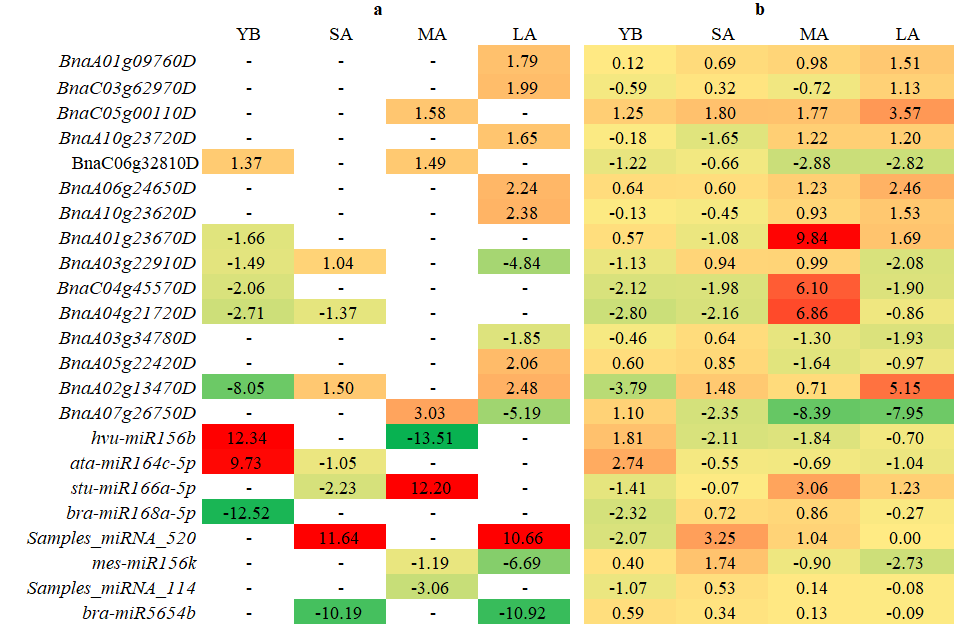

Supplement: Supplementary file 12 — Validation for DEGs and DE-miRNAs by qRT-PCR. a, Result from RNA-seq and miRNA-seq. b, Result from qRT-PCR. The numbers are log2X-normalized ratio values. Red color represents higher gene expression levels. Green color corresponds to lower gene expression levels. The blocks without a numerical value indicate the gene expression was not detected by RNA-seq or miRNA-seq. (JPG 110 kb) [file 12864_2018_5203_MOESM12_ESM.jpg]

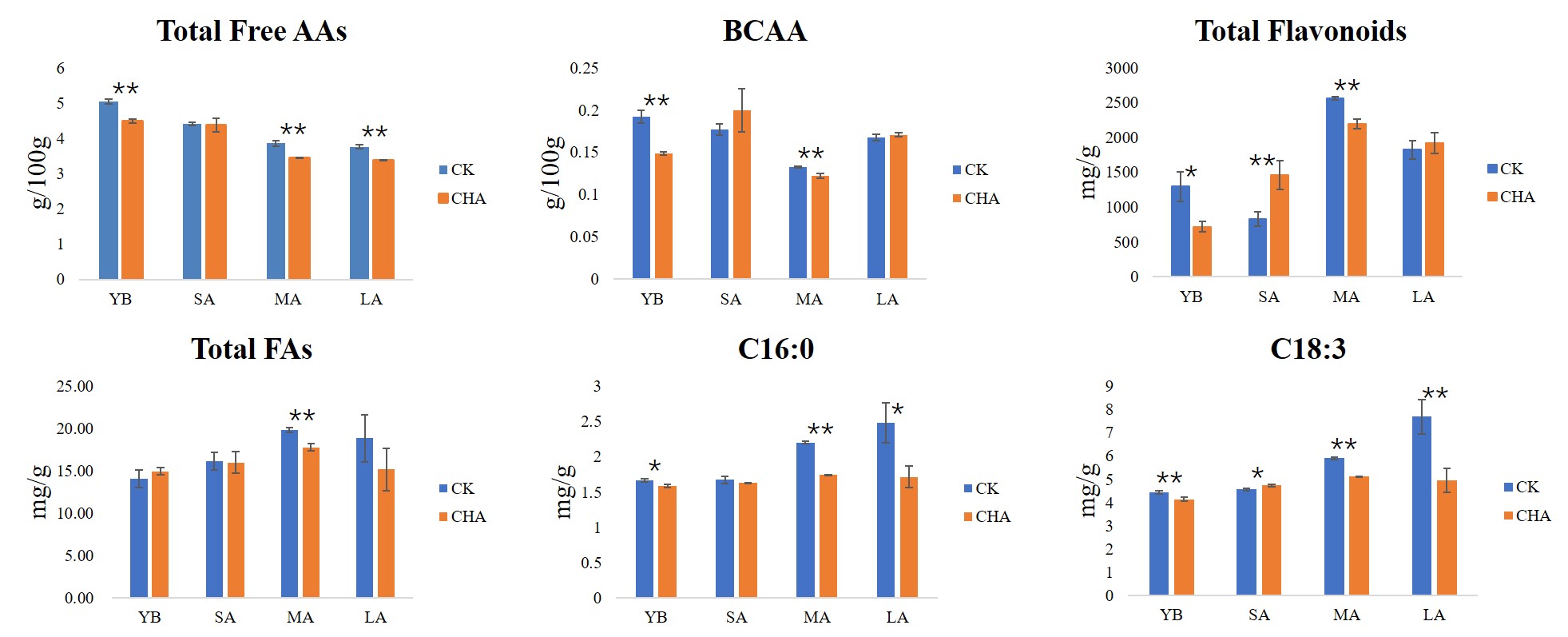

Supplement: Supplementary file 13 — Analysis of free amino acids, total flavonoids and fatty acids of the control and CHA materials. The values indicate means ± s.d., n = 3, *P < 0.05, **P < 0.01, by Student’s t test. (JPG 163 kb) [file 12864_2018_5203_MOESM13_ESM.jpg]

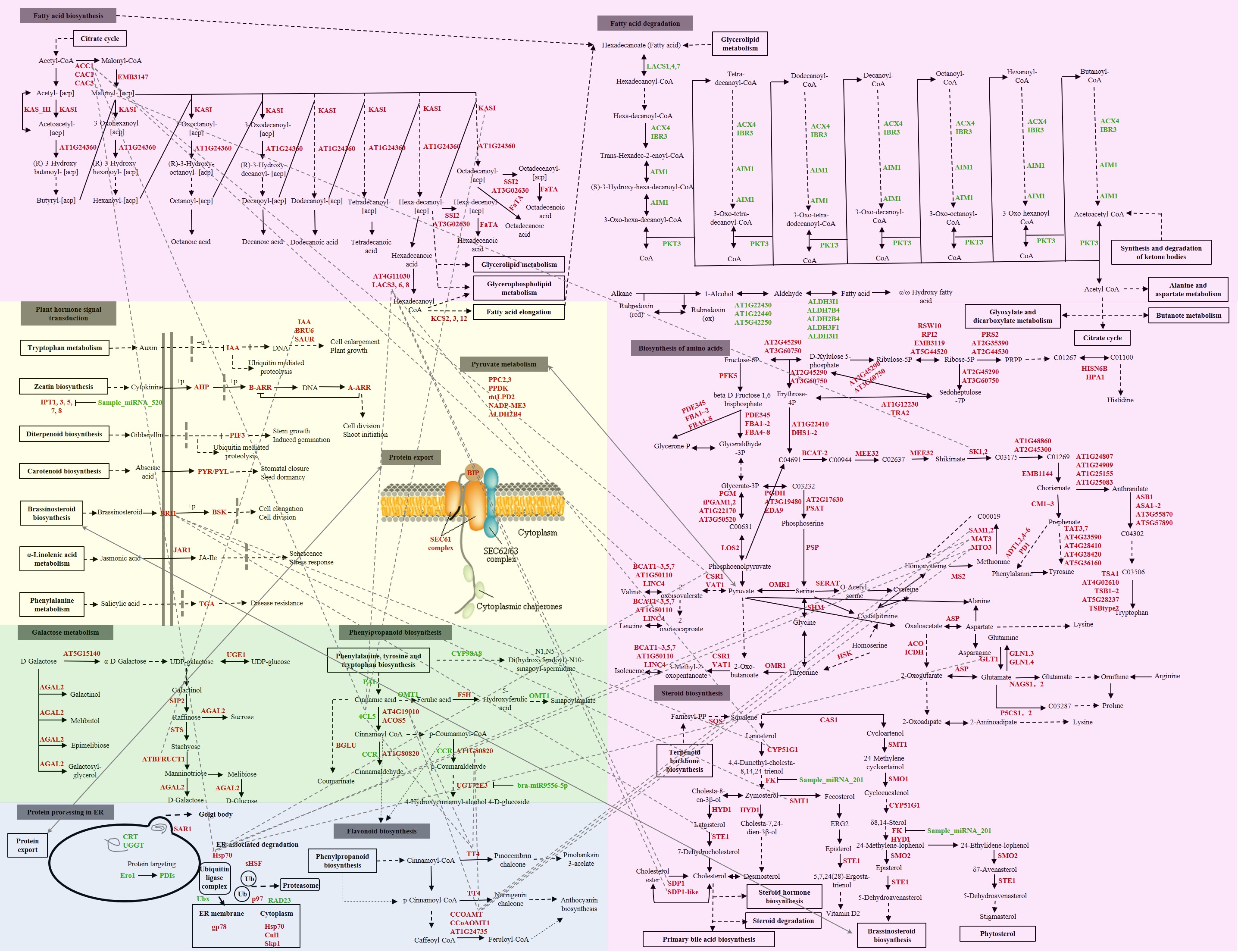

Supplement: Supplementary file 15 — The putative metabolic pathway for male sterility induced by SX-1. Red letters indicate down-regulation, and green letters indicate up-regulation. Gray dotted line means interaction between these two genes. Gray dotted arrow means there are many steps between the two metabolites. Gray two-way arrow means the same metabolite or pathway. Pathways located in the light blue background indicate the main events at the YB stage, light green for SA stage, light red for MA stage and light purple for LA stage respectively. (JPG 1163 kb) [file 12864_2018_5203_MOESM15_ESM.jpg]
